# The RNA binding activity of the first identified trypanosome protein with Z-DNA-binding domains

**DOI:** 10.1038/s41598-019-42409-1

**Published:** 2019-04-11

**Authors:** Najmeh Nikpour, Reza Salavati

**Affiliations:** 1 Institute of Parasitology, McGill University, Quebec, H9X3V9 Canada; 2 Department of Biochemistry, McGill University, McIntyre Medical Building, 3655 Promenade Sir William Osler, Montreal, Quebec H3G 1Y6 Canada

## Abstract

RNA-binding proteins play a particularly important role in regulating gene expression in trypanosomes. A map of the network of protein complexes in *Trypanosoma brucei* uncovered an essential protein (Tb927.10.7910) that is postulated to be an RNA-binding protein implicated in the regulation of the mitochondrial post-transcriptional gene regulatory network by its association with proteins that participate in a multi-protein RNA editing complex. However, the mechanism by which this protein interacts with its multiple target transcripts remained unknown. Using sensitive database searches and experimental data, we identify Z-DNA-binding domains in *T*. *brucei* in the N- and C-terminal regions of Tb927.10.7910. RNA-binding studies of the wild-type protein, now referred to as RBP7910 (RNA binding protein 7910), and site-directed mutagenesis of residues important for the Z-DNA binding domains show that it preferentially interacts with RNA molecules containing poly(U) and poly(AU)-rich sequences. The interaction of RBP7910 with these regions may be involved in regulation of RNA editing of mitochondrial transcripts.

## Introduction

One of the most intriguing features of *Trypanosoma brucei*, a unicellular kinetoplastid parasite, is its unique way of producing energy in different life cycle stages^1^. In the insect vector, the single mitochondrion in *T*. *brucei* undergoes extensive morphological alterations and changes in gene expression. One example of the regulation of mitochondrial gene expression is the insertion and deletion of uridine nucleotides during a unique post-transcriptional editing process that is restricted to kinetoplastids^2,3^.

Editing relies on mitochondrial-encoded small RNAs known as guide RNAs (gRNA) which contain the information needed for the proper addition of uridines to or deletion from pre-mRNAs to produce edited-mRNAs, which are translated into essential protein subunits of the respiratory chain. Additionally, gRNAs have (U)-tails at the 3′ end that are added post-transcriptionally by a mitochondrial terminal uridylyl transferase (TUTase)^4,5^. Upon binding to the pre-mRNA, the gRNA 3′ (U)-tail interacts with the purine-rich region upstream of the editing site (ES). This interaction, which stabilizes the gRNA-pre-mRNA duplex, has been suggested to be a possible function of the gRNA 3′ (U)-tail^4,6,7^.

In addition to the editosome or RNA editing core complex (RECC), which catalyzes the enzymatic steps of the editing process^8,9^, other dynamic multi-protein complexes have been identified. These include the RNA-editing mediator complex (REMC), guide RNA binding complex (GRBC), and the polyadenylation mediator complex (PAMC), collectively known as the RNA editing substrate-binding complex (RESC)^3,10–12^. The components of these complexes are essential for mitochondrial RNA editing and maturation of edited RNAs prior to translation. Some of the proteins in these complexes are needed for the processivity of the editing reaction and RNA utilization during this process^13,14^. Multiple proteins are transiently associated with the editosome or editing substrates to ensure accurate and efficient editing, and to influence the stability and abundance of mitochondrial RNAs. Some RNA-binding proteins (RBPs) bind mRNAs (pre-edited and edited transcripts) and/or gRNAs with various binding affinities^15,16^, or stabilize the gRNA conformation during the editing process^11,15,16^. For example, the paralogues GRBC1 and GRBC2 form a stable heterotetramer with a α2/β2 configuration in the core of MRB1 and help stabilize the gRNA^11,17^ by protecting the gRNA population from nucleolytic degradation^18^. gRNAs exhibit different primary sequences, but they share common secondary structures composed of two stem-loops and a 3′ oligo (U)-tail^6,19^.

The gRNA-binding proteins MRP1/2 bind gRNA via non-sequence-specific electrostatic interactions^20,21^. On the other hand, other proteins show gRNA-binding ability mediated by the U-tail of the gRNA, such as KREPA4 in the RECC^22^, and the multifunctional RNA-binding protein RBP16^23^. The U-tail is likely a single-stranded molecule with a partially helical arrangement^24^ that helps stabilize the gRNA/mRNA duplex^18,25^. Pentatricopeptide repeat RBPs represent another group of RBPs in *T*. *brucei*, and include kinetoplastid polyadenylation factor 1, 2 (KPAF1/2)^26^, which functions in the synthesis of the long 3′ tail of edited mRNAs, and KPAF3^27^, which is more crucial in the process of selecting pre-mRNAs for adenylation rather than uridylation before entering the editing pathway.

We previously reported a mitochondrial protein (Tb927.10.7910) that interacts with REMC5A and TbRGG2, subunits in the RESC, in an RNA-dependent manner^28^. Down-regulation of this protein indicated its essential role in cell viability via editing of the apocytochrome b mRNA in the insect form of *T*. *brucei*. Another study also showed RNA-dependent interactions of the protein in tandem affinity purification of multiple subunits of the RESC^12^. Recently, Tb927.10.7910 was described as lacking specific motifs and to be part of a complex named the PPsome^29^. This study illustrated the link between the PPsome and the RESC and its role in converting the mitochondrial transcription-defined 5′ terminus into a monophosphorylated state.

In the present study, we identified two winged helix-turn-helix (HTH) structured Z-DNA- binding domains in Tb927.10.7910, resembling a conserved family of proteins with Z-DNA-binding domains (ZBPs), known to bind specifically to Z-DNA and/or Z-RNA^30,31^. *In vitro* RNA-binding and competition assays revealed the RNA-binding activity of the recombinant protein (hereafter called RBP7910), which recognizes multiple mitochondrial RNA classes containing poly(U) and poly(AU)-rich sequences through the nucleic acid recognition core of its Z-DNA-binding domains.

## Results

### Identification of potential Z-DNA-binding domains in RBP7910

**Figure 1:**
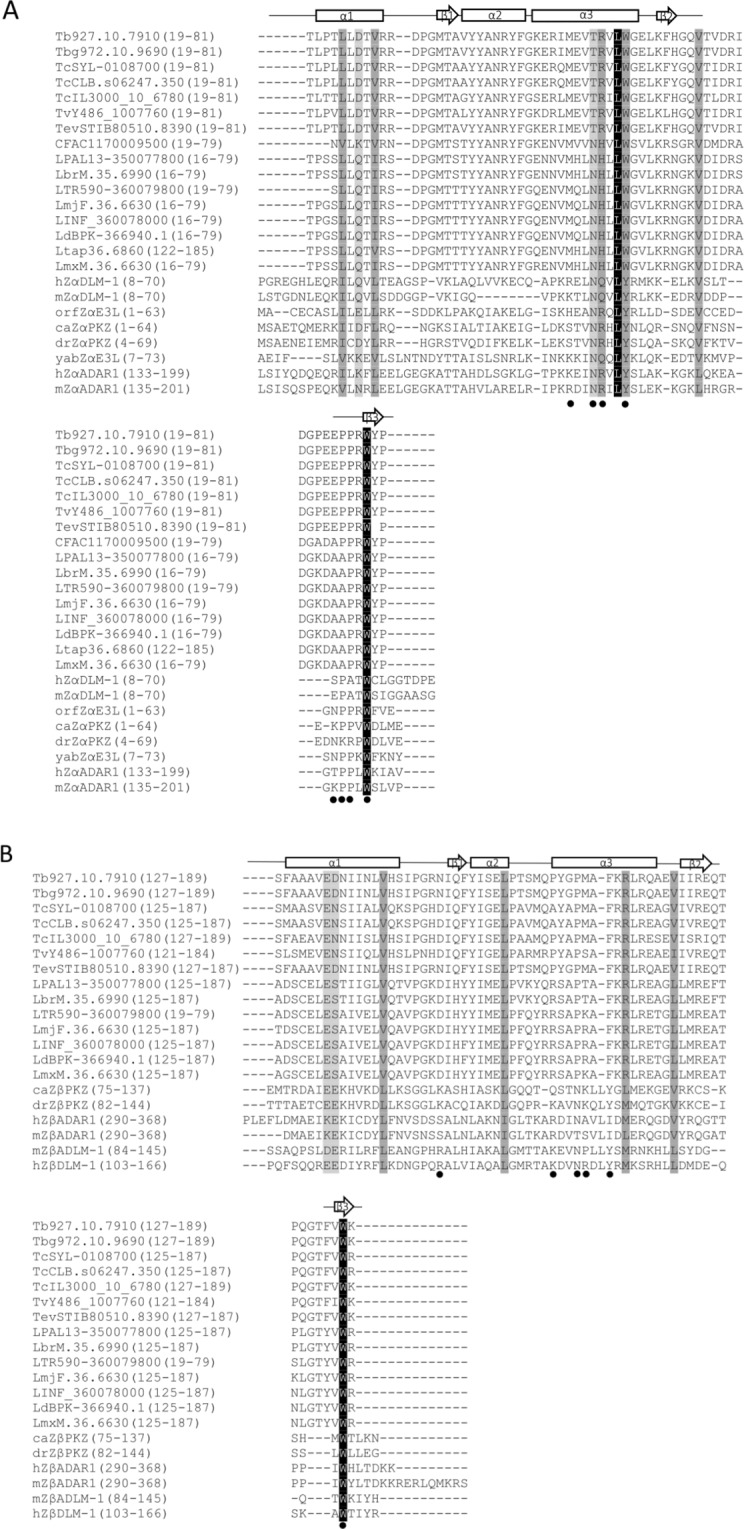
Multiple amino acid sequence alignment of predicted N- and C-terminal ZBDs of RBP7910 with Zα and Zβ domains of ZBPs, respectively. (**A**) Sequence conservation of the N-terminal ZBD of RBP7910, its orthologues from various kinetoplastid species, and Zα of other ZBPs and (**B**) sequence conservation of the C-terminal ZBD of RBP7910, its orthologues from various kinetoplastid species, and Zβ of other ZBPs. Predicted secondary structure of ZBD is indicated above the sequence of the first and second Z-domains of RBP7910. The α-helices are represented by tubes and β-strands by bold arrows. hZαADAR1 and hZβDLM-1-DNA interactions are marked with black circles. Shading from black to white corresponds to the degree of the amino acid conservation. Black shaded residues represent 100% identity. Numbers in parentheses correspond to the domain boundaries within the respective protein sequence. The sequences are as follows: RBP7910, kinetoplastid species, including *T. b. gambiense, T. cruzi Sylvio, T. cruzi CL, T. congolense, T. vivax, T. evansi, Crithidia fasciculata, Leishmania panamensis, Leishmania braziliensis, Leishmania tropica, Leishmania major Friedlin, Leishmania infantum, Leishmania donovani, Leishmania tarentolae, Leishmania Mexicana*, and for Zαs; DLM-1 from *Homo sapiens* in hZαDLM-1 and *Mus musculus*, mZαDLM-1; E3L from orf virus in orfZαE3L and yabZαE3L from Yaba-like disease virus; PKZ from goldfish, caZαPKZ and drZαPKZ in zebrafish; ADAR1 from *Mus* musculus, mZαADAR1, and hZαADAR1 in *Homo* sapiens. Zβs include goldfish PKZ, caZβPKZ and zebrafish PKZ, drZβPKZ; ADAR1 in hZβADAR1 from *Homo* sapiens and *Mus* musculus, mZβADAR1; DLM-1 in *Mus* musculus, mZβDLM-1, and hZβDLM-1 from *Homo* sapiens.

The conventional sequence search methods BLAST^32^ and PSI-BLAST^33^ were used to interrogate potential biological functions of RBP7910 based on homologous proteins identified in a sequence search. The similarity search tools based only on sequence returned no relationships to proteins with known function. In an alternative strategy, we used HHpred, a highly sensitive method for searching for more remotely homologous relationships^34^. Using an improved version of profile-sequence comparison, Profile Hidden Markov Models (HMMs), HHpred predicted two potential Z-DNA-binding domains in the N- and C-terminal regions of RBP7910. The N- or C-terminal sequences were input into HHpred or I-TASSER server instead of the complete sequence^35,36^ and dramatically improved the accuracy of the predicted function and secondary structure of each domain. The secondary structure prediction showed three-helix bundles and three β-sheets with an αβααββ topology for both domains. Similar α/β HTH architecture, consisting of three α-helices and three β-strands, has been observed in Z-DNA-binding proteins (ZBPs)^30,37,38^. Multiple sequence alignment of N- and C- terminal domains of RBP7910 with some of its orthologs and corresponding domains of known ZBPs is shown in Fig. 1. To date, four protein families with one or two tandem Z-DNA-binding domains have been identified: ADAR1, DLM-1 or ZBP1, a protein kinase from fish containing a Z-DNA-binding domain (PKZ) and the viral protein E3L^38–41^. ADAR1, DLM-1, and PKZ contain two Z-DNA-binding domains (Zα and Zβ, respectively), whereas E3L has one Zα domain. The nucleic acid binding activity of the Zα and Zβ domains of different ZBPs has been widely studied. The Zα domain exhibits a higher level of sequence conservation than the Zβ domain. Crystallographic data for ZBPs showed that residues from α3 and the β2/β3 wing region serve as the nucleic acid binding interfaces^31,38,42^ (Fig. 1). The N-terminal domain of RBP7910 also shows a greater sequence conservation than the C-terminal sequence, particularly at the nucleic acid-contacting interfaces.

### RBP7910 has higher affinity for gRNAs than mRNAs

We determined the binding affinity of recombinant RBP7910 for radiolabeled gRNAs and pre-edited and edited mRNAs using electrophoretic mobility shift assay (EMSA) to assess the RNA-binding ability of predicted Z-DNA-binding domains of RBP7910. RNA substrates including (gA6[14])^43^, A6U5 pre-mRNA^6^, edited A6U5 (deletion of 3Us), CYb gRNA (gCYb [558] USD-2A-42nt)^44^, native CYb gRNA (gCYb [558])^45^, CYb pre-mRNA^46^, and CYb edited-mRNA^47^ were *in vitro* transcribed and labeled with [α- ^32^P] either during transcription or after transcription at the 3′ end of the mRNA. Despite the detection of a protein-RNA complex between recombinant RBP7910 and gA6[14] and pre-and edited CYb mRNAs, we did not detect binding between this protein and the A6 pre-mRNA or any CYb gRNA variant (data not shown).

**Figure 2:**
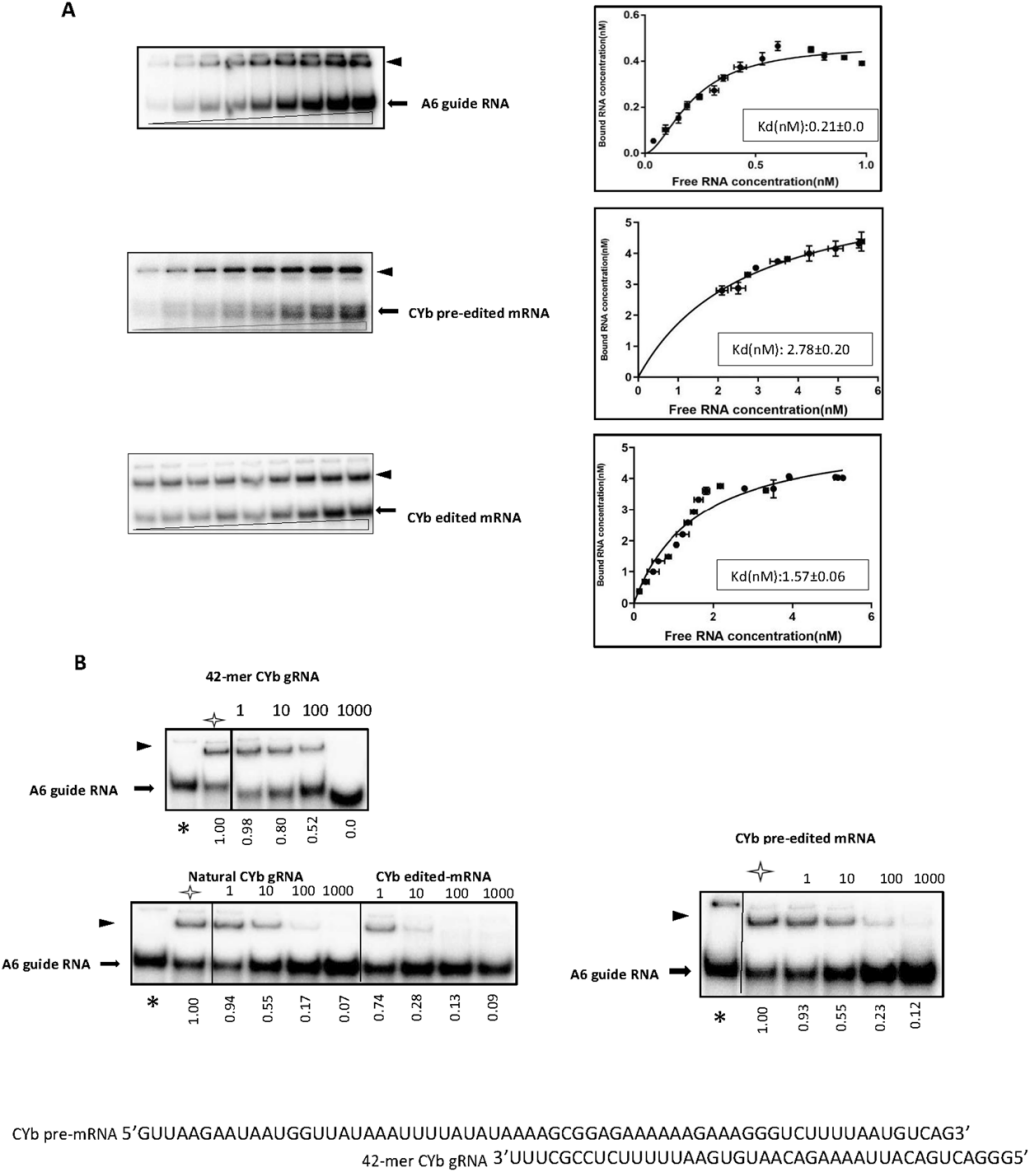
Examination of the RNA-binding activity of RBP7910 using EMSAs. (**A**) Gel mobility shift assays show the binding of 40 nM of the recombinant protein to increasing concentrations of different RNA substrates. The wedges show the increasing concentrations of ^32^P-labeled RNAs (0.1–1.2 nM, gA6[14]; 5–30 nM, pre-edited CYb; and 0.5–10 nM edited CYb), and shifted bound protein-RNA complexes are marked with black triangles. Bound and free RNA concentrations from the experiments shown in panel A were used to estimate the binding activity of RBP7910 to each RNA substrate (left panel). The saturation binding curve was obtained using none-linear regression analyses of five individual experiments for each substrate. Calculated Kd ± SD values in nanomolar units are shown for each labeled RNA substrate. (**B**) Competition assays verified the better affinity of the protein to gA6[14] to other guides and mRNAs. Competition assays were done by incubation of a fixed concentration of purified protein and labeled gA6[14] in the absence and presence of increasing concentrations of unlabeled competitors (gCYb RNA variants, pre-edited, and edited CYb mRNAs). Asterisk indicates the input labeled RNA in the absence of the protein and the white star shows the labeled RNA with protein in the absence of the competitor RNA. Numbers above the panels indicate the fold excess of the unlabeled RNA competitors and numbers below of each panel is the shift percentage in the presence of competitor RNAs normalized to the shift in the absence of a competitor $(whitestar)$ . The name of unlabeled RNA substrate used for each assay is indicated above each panel along with the complete sequence under each panel.

The incubation of a fixed amount of RBP7910 with increasing concentrations of radiolabeled gA6[14] or pre-edited and edited CYb mRNAs resulted in the formation of a slowly migrating protein-RNA complex (Fig. 2A). The Kd for the interaction of recombinant RBP7910 with each labeled RNA substrate was estimated from five individual experiments, and the results were analyzed using a non-linear regression model. As shown in Fig. 2A, the Kd value for the wild-type (WT) protein interacting with the U-tail-bearing A6 guide RNA substrate was determined to be 0.21 ± 0.01 nM; 95% CI:0.18, 0.27, indicating a significantly higher affinity for this target than for the edited CYb mRNA (1.57 ± 0.06 nM; 95% CI:1.25, 1.97) and pre-edited CYb mRNA (2.78 ± 0.20 nM; 95% CI:2.09, 3.66) substrates.

To support these results, we performed competition experiments using labeled gA6[14] RNA, unlabeled CYb gRNA variants and CYb mRNAs as competitors. The natural gCYb RNA with the U-tail competed for binding between RBP7910 and gA6[14] RNA 10 times better than the 42-mer CYb gRNA without the U-tail (Fig. 2B). The same concentrations of natural gCYb RNA and pre-CYb mRNA (10-fold molar excess of unlabeled RNAs) reduced binding of the labeled RNA by 50%. However, the edited CYb mRNA, which contains approximately double the number of Us compared to the pre-edited CYb RNA, competed for binding more efficiently by producing a 30% reduction in binding of labeled gA6[14] RNA at a 10-fold molar excess of unlabeled edited CYb mRNA. We performed competition experiments, discussed in the next section, to examine the specificity of binding for each substrate and to define the role of structural features of the gRNA in the RBP7910-gA6[14] RNA interaction.

### gRNA and mRNA-binding specificity of RBP7910

**Figure 3:**
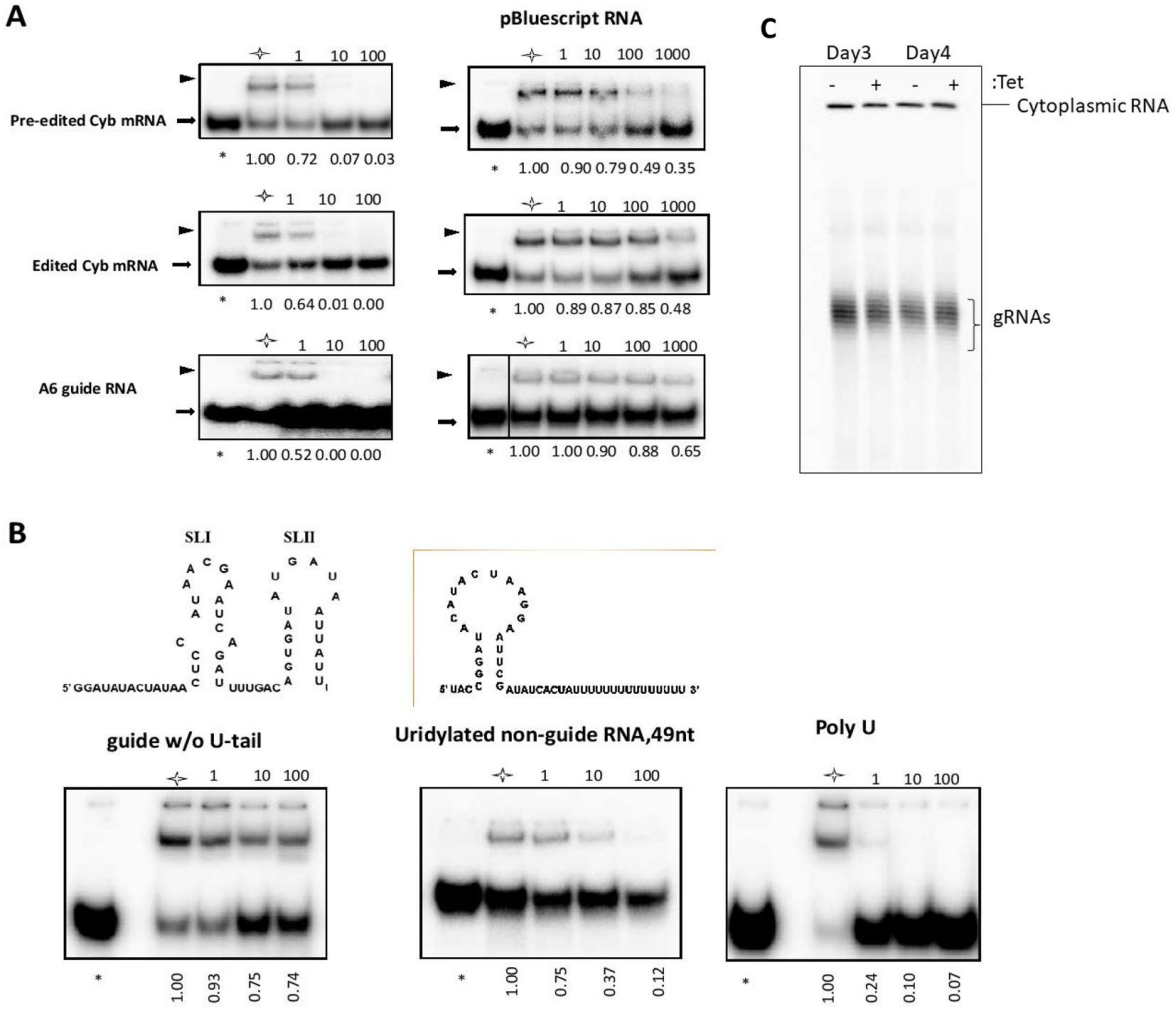
Competition assays to determine the binding specificity of RBP7910 for CYb pre-edited and edited RNAs and gA6[14] RNA. (**A**) RBP7910 protein was individually incubated with labeled pre-edited CYb, edited CYb, and gA6[14] RNAs in the absence and presence of increasing concentrations of each related unlabeled RNAs as a competitor. Labelling of the panels in this figure follow the same order as Fig. 2. Left panel, same as A; except that it uses a different competitor, pBlueScript RNA. (**B**) Competition assays to determine the role of gRNA oligo (U)-tail and stem-loop structure in gRNA binding. Three different competitors were examined to clarify the role of the oligo (U)-tail and the secondary structure of the gRNA in RBP7910 binding including gA6 RNA without the oligo (U)-tail, uridylated non-guide RNA with one predicted stem-loop and an oligo (U)-tail, and poly-U RNA. A fixed concentration of RBP7910 was incubated with labeled gA6[14] in the absence and presence of increasing concentrations of unlabeled competitors. (**C**) The effect of RBP7910 RNAi silencing on gRNAs. RNAs from Tet-induced and uninduced RBP7910 RNAi 3d and 4d post-induction were capped with [α-^32^P] GTP by the recombinant guanylyltransferase enzyme. The population of small gRNA molecules was resolved as a ladder of bands on a denaturing 8% acrylamide/7 M urea gel (bottom panel). A cytosolic RNA (top panel) is simultaneously labeled by this reaction and is shown as a loading control.

Gel shift assays were conducted to examine the specificity of binding of each substrate using radiolabeled substrates in the presence of increasing concentrations of unlabeled RNAs, including gA6[14] and pre- and edited CYb mRNAs. Unlabeled homologous RNAs reduced binding at the same molar ratios of labeled RNAs and eliminated RBP7910-labeled RNA interactions at 10-fold molar excess concentrations (Fig. 3A, left panel). We also examined competitive binding using a heterologous 92nt pBlueScript RNA at up to a 1000-fold excess (Fig. 3A, right panel) and observed negligible competition for binding of RBP7910 with the CYb mRNA and A6 gRNA.

We also assessed the affinity of RBP7910 for the poly (U)-tail of the gRNA by performing a competition assay using unlabeled gA6[14] RNA lacking the U-tail with gA6[14] RNA (Fig. 3B). While an equimolar ratio of the unlabeled gA6[14] RNA with the U-tail completely competed for binding of labeled gA6 [14] RNA (Fig. 3A), a 100-fold molar excess of unlabeled gA6[14] RNA lacking the U-tail only reduced complex formation by 25%. This result indicates the importance of the U-tail in the RBP7910-gRNA-binding process. We confirmed this finding using unlabeled poly U as a competitor and showed that it competed with the bound complex at an equimolar ratio of unlabeled poly U and labeled gA6[14] RNA (Fig. 3B).

Thus, we used a uridylated non-guide RNA (49 nt) as the competitor to more comprehensively investigate the contributions of stem-loop elements (secondary structure) and the U-tail in the RBP7910-gRNA interaction (Fig. 3B). This RNA has a shorter poly U-tail (15 nt) and only one stem-loop compared to the gA6[14] RNA. The non-guide RNA was more efficient in competing the RBP7910-gA6[14] complex than gA6[14] RNA lacking the U-tail, but was still 10 times less efficient than gA6[14] RNA. This result indicates indispensable roles for the oligo U-tail and the secondary structure of the gRNA in the RBP7910-gRNA interaction, although again suggesting that the oligo U-tail is the main determinant.

In light of the high affinity of RBP7910 for gRNA, we asked if RBP7910 possesses a general gRNA stabilizing activity during the RNA editing process, similar to the gRNA-binding proteins GRBC1 and GRBC2^11^. We compared the total gRNA population between Tet-induced and uninduced cells expressing a RBP7910 knock-down RNAi construct 3 and 4 days post-induction^28^ using guanylyl transferase labeling to determine the contribution of RBP7910 to the stability of the total gRNA population (Fig. 3C). No prominent changes were observed in the levels of gRNAs between induced and uninduced samples. Therefore, the major gRNA-binding activity of RBP7910 is not related to stability of gRNAs, and the gRNA-binding activity of the protein is part of the general RNA-binding activity of RBP7910. However, we cannot exclude the possibility of a transcript-specific effect of RBP7910 on gRNA stability in the absence of data on individual gRNAs.

### RBP7910 shows distinct affinity for AU-enriched sequences

Mitochondrial mRNAs and gRNAs are AU-rich transcripts with multiple biological functions. According to Brown and colleagues^48^, AU elements in the pre-edited CYb mRNA function as the primary assembly point for the editosome machinery. Following the binding of the gRNA to the pre-edited CYb mRNA, editing factors are transferred to the AU elements of the gRNA. Similarly, another study showed the importance of the AU sequence for formation of the pre-edited/gRNA duplex using A to C point mutations within the gRNA-binding site that interfered with the formation of the pre-edited/gRNA duplex^49^ and reduced editing by 80%.

Another AU structure in mitochondrial transcripts is the long AU-tail, a post-editing AU extension of the primary short A-tail of pre-edited transcripts. The long AU-tail is a hallmark of the translation process of fully-edited transcripts^26^. In addition to the general factors involved in synthesis of the long AU-tail, such as RET1, KPAP1, and KPAF1, other RBPs selectively affect the stability of mitochondrial mRNAs containing AU-tails and activate their translation at the insect life stage^50^.

**Figure 4:**
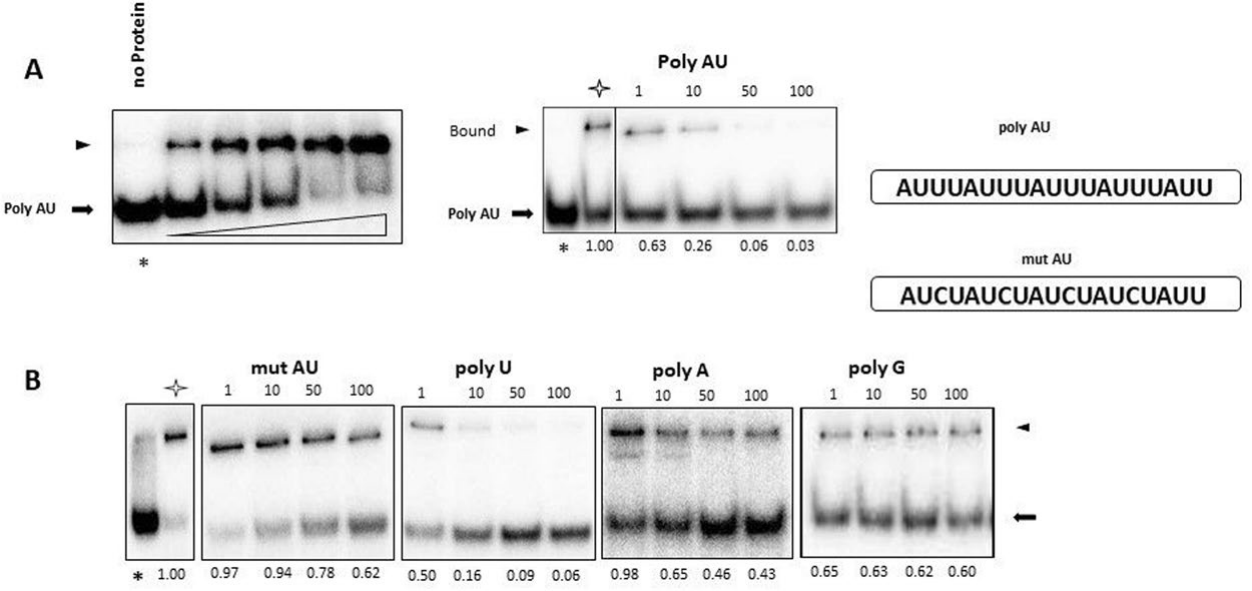
Competition assays to determine the affinity binding and specificity of RBP7910 to the labeled poly AU sequence. (**A**) Titration of RBP7910 protein over 1 nM concentration of labeled poly AU RNA. The first lane is the labeled poly AU in the absence of the protein. The protein-RNA bound complexes are shown by a black triangle. Middle panel, RBP7910 RNA binding specificity was checked using unlabeled homologous poly AU competitor in a competition assay. RBP7910 at 40 nM was incubated with labeled poly AU in the absence and presence of increasing concentrations of unlabeled competitor. Asterisk indicates the input labeled RNA in the absence of the protein and the white star shows the labeled RNA with protein in the absence of the competitor RNA. Numbers above the panels indicate the fold excess of the unlabeled RNA competitor and numbers below of each panel is the % shift in the presence of competitor RNA which normalized to the shift in the absence of a competitor (**B**), same as A; except than using different competitors. Competitor RNAs mentioned above each panel.

Considering the RNA-binding activity of RBP7910, we next questioned the potential AU sequence-binding affinity of RBP7910. We labeled a poly AU sequence previously found to be enriched in 3′ untranslated region of many trypanosomatid genes^51^. Incubation of increasing concentrations of RBP7910 with a fixed amount of labeled poly AU RNA led to the formation of a RNA-protein complex. The specificity of the protein-RNA interaction was confirmed in a competition assay using the unlabeled RNA (Fig. 4A).

In light of the importance of the AU sequence during the editing process and for duplex formation of gRNA/pre-edited mRNA^48,49^, we assayed the interaction of RBP7910 with a modified poly AU sequence containing U to C substitutions. The ability of this U to C-substituted poly AU RNA to compete for binding was largely abolished compared to the poly AU substrate (Fig. 4B).

We tested the abilities of poly U, poly A, and poly G RNAs to compete with the RBP7910 - poly AU interaction as a method to determine whether RBP7910 prefers poly AU or poly U as substrate. Poly U RNA was the most competitive substrate, as it decreased complex formation by 50% at an equimolar concentration, while poly A and poly G RNAs were similar to the U to C-substituted poly AU RNA, as shown above.

Based on these results, we conclude that RBP7910 binds to AU-containing RNAs. However, we were unable to determine whether RBP7910 binds to the internal AU sequence of the mitochondrial substrates (gRNAs and mRNAs) or the poly AU-tail of mitochondrial transcripts. Although we have shown binding of RBP7910 to the AU sequence, we could not determine if this interaction is purely sequence specific or mediated by the secondary structures of the sequence. Considering the binding preference of RBP7910 for the poly AU sequence, we propose that this protein is likely to be involved in RNA editing or translation of mRNAs containing an AU-tail.

### Functional analysis of the RNA-binding activity of RBP7910 using structure-based mutagenesis

Following experimental establishment of the RNA-binding activity of RBP7910, we were interested in identifying residues that affect RNA binding based on sequence and structural alignments. Structural predictions and sequence analysis of RBP7910 identified two putative Z-DNA-binding domains in the N- and C-termini of RBP7910. The Z-DNA-binding domain family belongs to the superclass of protein with WHTH domains, and this superclass is largely present in the DNA-binding domain of prokaryotic transcription factors and some eukaryotic transcription factors^52^. This domain specifically recognizes the Z-form of DNA/RNA molecules in a conformation-specific manner. Because RBP7910 and ZBPs exhibit a similar fold, we examined whether they also shared the same nucleotide-binding interface. Zα and Zβ are structurally homologous domains with a similar arrangement of α helices and β sheets (αβααββ), with the exception of the presence of one extra helix (α4) in ZβADAR1, which is mostly involved in dimerization of the protein^37^.

The RNA-binding function of RBP7910 was probed by replacing candidate RNA-contact residues in the N- and C- terminal domains of RBP7910 with alanine. Sequence comparisons of different ZBPs suggested the presence of a common nucleic acid recognition core containing hydrophobic and positively charged amino acids in the α3 core and the β2/β3 wing^53^. As shown in Fig. 1, Asn173, Tyr177, and Trp195 of hzαADAR1 are the most conserved core residues in ZBPs^30,54^. These residues are also conserved in the hZβDLM-1/Z-DNA complex^53^, although with a different hydrogen bonding pattern. Furthermore, ZBPs contain one or two proline (P192-P193 of hzαADAR1) residues that contribute to the Zα DNA-binding activity via hydrophobic interactions. These proline residues are usually located adjacent to a polar residue such as Thr or Asn, which interacts with DNA through water-mediated hydrogen bonds^30,38,55^. No equivalent residue for the Pro or Thr residues of Zα are present in Zβ domains.

A few mutagenesis studies have investigated the Z-DNA/RNA-binding activities of ZBPs. For instance, alanine substitution for Asn173 and Tyr 77 in hZαADAR1^54,56^ or the corresponding residues in mZαDLM-1 and mZβDLM-1^57^ eliminated the DNA-binding ability of each domain without altering protein stability.

The N-terminal domain of RBP7910 showed a high level of conservation for residues in the nucleic acid recognition core of ZαZBPs (Fig. 1). Thr52 and Trp56 replace Asn173 and Tyr177 from hZαADAR1 in the third predicted helix, although Thr52 is conserved among *Trypanosoma* genera and Trp56 is conserved in kinetoplastids. Arg53 is also shared among *Trypanosoma* genera, Pro76 in *Trypanosoma* genera and *C*. *fasciculata*, and Pro77 and Trp79 are conserved in kinetoplastids. Different amino acids from the N- and C-terminal domains of RBP7910 were selected for mutagenesis studies based on (1) conservation of amino acids located in the recognition core of ZBPs, (2) previously reported point mutations affecting nucleic acid-binding activity of ZBPs, and (3) avoiding residues previously reported to be crucial for the protein stability.

A gel retardation assay was employed to assess the effects of each point mutation on the binding of ^32^P-labeled gA6 RNA to RBP7910. Selected amino acids in the N-terminal domain and the third helix (α3) of RBP7910 were Thr52, Arg53, and Trp56; Pro76, Pro77, Trp79 were selected from the β2/β3 wing. Because of the lower conservation of the Zβ domain of ZBPs and kinetoplastids, only Pro164, Phe167, and Trp188 from the Zβ recognition core were chosen for mutagenesis analysis of the second predicted Z-DNA-binding domain of RBP7910.

**Figure 5:**
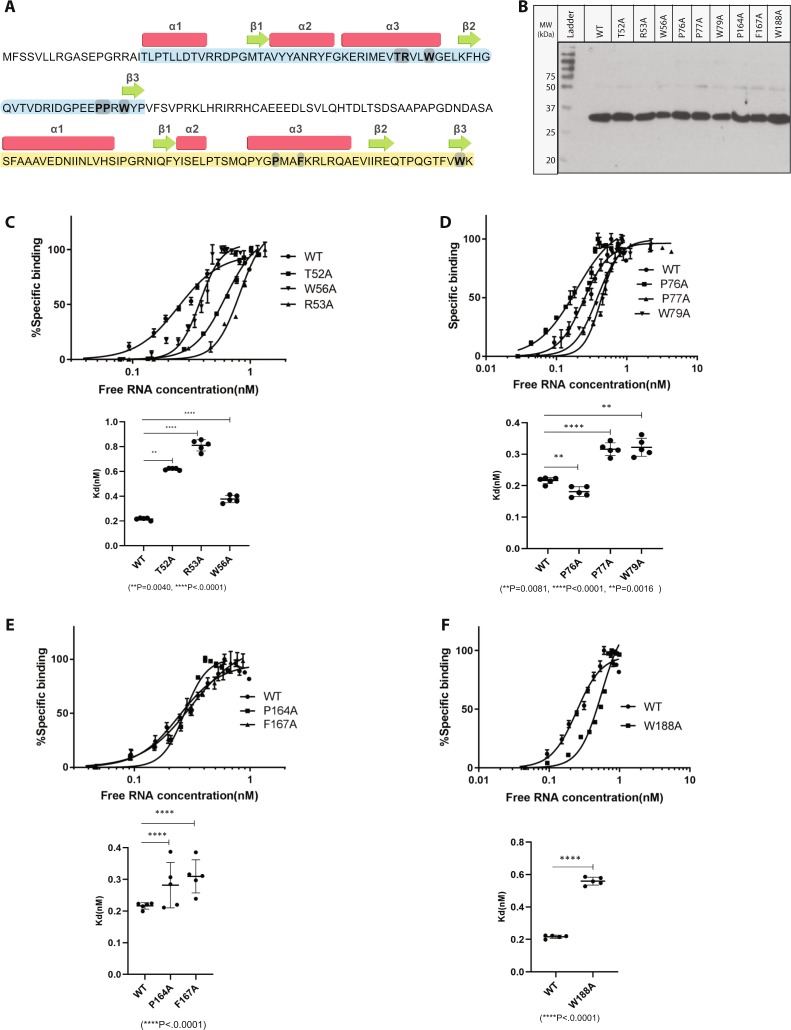
gA6[14] RNA-binding activities of RBP7910 point mutations measured by gel shift mobility analysis. (**A**) The complete amino acid sequence of RBP7910 is shown with the predicted N- and C-terminal ZBDs in blue and yellow, respectively. The α-helices and β-strands of the predicted RNA recognition core in ZBDs are represented by boxes and by arrows, respectively. The point mutations used in this study are in bold and shaded. (**B**) Purified, recombinant his-tagged WT RBP7910 and mutant proteins were analyzed by 12% SDS-PAGE. The molecular weight marker is shown on left side. Binding activities of point mutations selected from the α3 (**C**) and wing region (**D**) of the predicted ZαRBP7910, and the α3 (**E**) and wing region (**F**) of the predicted ZβRBP7910. Binding activities of mutants from each region were quantified using a nonlinear curve fitting method, as it was done previously for the WT RBP7910. Kd values of each point mutation were calculated and compared to the Kd of WT RBP7910. Data are presented as mean ± SD unpaired two-tailed *t*-test.

RPB7910 point mutations affected RNA-binding affinity of the protein to varying extents. The T52A, R53A, and W56A mutants exhibited a reduced binding affinity for the A6 gRNA compared to the WT protein, with Kd values of 0.61 ± 0.008 nM; 95% CI:0.53,1.52, 0.81 ± 0.04 nM; 95% CI:0.70,2.07, and 0.37 ± 0.02 nM; 95% CI:0.33,0.54, respectively (Fig. 5C). The P76A mutant located in the β2/ β3 wing region of RBP7910 differentially influenced the RNA binding activity of RBP7910 compared to the P192A substitution in hZαADAR1^54^. Previous mutagenesis studies showed a negative effect of P192A on DNA-binding activity of hZαADAR1, while the P67A substitution, with a Kd value of 0.18 ± 0.01 nM; 95% CI:0.13,0.31, showed 1.2-fold better binding affinity than WT RBP7910 (Fig. 5D). However, similar to P193A, the P77A mutant with a Kd of 0.30 ± 0.02 nM; 95% CI:0.23,0.36 exhibited 1.3-fold lower affinity than WT RBP7910. As expected from the central Z-DNA-binding role of the conserved tryptophan in the β3 strand of other ZBPs, the W79A mutant showed a Kd of 0.32 ± 0.02 nM; 95% CI:0.24,0.47, and 1.5-fold lower affinity than the WT protein. The core residues of ZβDLM-1 include N141, Y145, and W162, which mediate the interaction in hZβDLM-1/Z-DNA complex^53^. In addition to these residues, R142 of hZβDLM-1 seems to play a role similar to R174 of hZαADAR1 in Z-DNA recognition. Kd values for the P164A, F167A, and W188A mutants of RBP7910 were 0.27 ± 0.07 nM; 95% CI:0.22,0.46, 0.30 ± 0.05 nM; 95% CI:0.23,0.94, and 0.55 ± 0.02 nM; 95% CI:0.49,1.46, respectively, and these proteins exhibited 1.2, 1.5, and 2.9-fold lower affinity than the WT RBP7910 protein (Fig. 5E,F). Overall, these data support a mode of interaction similar to ZBPs, mediated by residues located in the predicted recognition core of RBP7910.

## Discussion

The data presented here identify the first Z-DNA-binding domain in a *T*. *brucei* protein with RNA-binding activity, which functions in mitochondrial RNA processing. The RNA binding activity of RBP7910 was suggested by its RNA-dependent interactions with REMC5A and TbRGG2 in the RESC^28^. This conclusion is supported by another study that detected RBP7910 through pull-down experiments of several individual members of the RESC^12^, while the RNA molecule mainly enforced these interactions. A recent study of biotinylated interacting partners of RBP7910 using a RB7910-BirA biotin ligase fusion protein^29^ confirmed REMC5A and TbRGG2 as the main interacting proteins in the RESC^28^. This work also showed interactions of RBP7910 with MERS1 NUDIX (nucleoside diphosphates linked to any moiety (x)) hydrolase, which with MERS2 PPR RNA-binding factor constitutes a 5′ pyrophosphohydrolase complex termed the PPsome. Therefore, we provide new insights into the role of RBP7910 in 5′processing of pre-edited transcripts as part of the PPsome. Identification of the PPsome purine-rich sites in 5′pre-edited transcripts with three Us at their 3′ end as the binding site of MERS2 along with the poly U binding affinity of RBP7910 suggests a role for RBP7910 in mitochondrial editing by engaging an RNA-dependent interaction of the PPsome with the RESC.

The high affinity of RBP7910 for U-rich and AU-rich RNA is consistent with Z-like steps found in RNA as r(U/ApA) dinucleotide repeats at key locations in single-stranded RNA regions and riboswitches^58–60^. Based on the results of the RBP7910 binding assays, in addition to the 3′oligo (U)-tail, the secondary structure of gRNAs is also important for the gRNA-binding activity of RBP7910. The secondary structure conformation of purine-pyrimidine repeats in DNA/RNA strands is the main factor responsible for the recognition of these molecules by ZBPs^59^. By considering the importance of the secondary structure for gRNA-binding by RBP7910 and the AU sequence binding preference of this protein, we suggest a crucial role for the secondary structure of the purine-pyrimidine AU-rich sequences for RBP7910 RNA-binding.

The interaction of Zα with the sugar-phosphate backbone of left-handed Z-DNA/RNA has been widely investigated^38,54^, suggesting that Zα binds to Z-DNA/RNA substrates using similar binding interfaces^31,61^. Compared to other ZBPs, the residues involved in nucleic acid recognition by RBP7910 are conserved but not identical, with few exceptions. However, alanine substitution point mutations of residues in the predicted binding interfaces only resulted in ~2–4 fold reduction in affinity of RBP7910 for RNA, lower than reported in other mutational studies of ZBPs^40,41,61^. One example of the Kd estimation is the Y177A substitution in ZαADAR1, which resulted in a 17.5-fold decrease in binding of a Z-DNA substrate compared to the WT protein^40^. It should be noted, however, that previous mutational analyses were performed based on the interaction of ZαADAR1 with DNA. We speculate that the discrepancy between the mutational effects is due to higher stability of the RNA-protein interaction. The ribose 2′-OH groups of RNA can make either direct or water-mediated hydrogen bonds with amino acids at the binding interfaces, and therefore single point mutations would not have a significant effect on RNA binding^31,62^.

In summary, mutational studies support the RNA-binding function of the recognition core in the Z-DNA-binding domains of RBP7910. Further experiments, such as the construction of RBP7910 Zα and Zβ truncations, will facilitate studies of the contribution of each domain to the RNA-binding activity of the protein. The nucleic acid binding activities of winged HTH domain-containing proteins have different biological implications in cells, such as the regulation of transcription, RNA biogenesis, translation, and immune responses. Similarly, the elucidation of the mode of RNA-binding activity in RBP7910 will be an interesting topic for future research to characterize possible regulatory roles of RBP7910 in mitochondrial RNA processing in *T*. *brucei*.

## Materials and Methods

### Database searches and sequence alignment

The RBP7910 sequence was analyzed for the presence of recognizable domains using HHpred^63^, resulting in the prediction of two Z-DNA-binding domains in the N- and C-terminal regions of Tb927.10.7910. The amino acid sequence of the N-terminal region of Tb927.10.7910, its orthologues in multiple kinetoplastid species (www.tritrypdb.org), and the Z-alpha (Zα) domain of ZαADAR1, DAI or ZBP1/ZαDLM-1, virus E3L (ZαE3L), and protein kinase containing a Z-DNA-binding domain (ZαPKZ) were aligned using Clustal Omega^64,65^. The same alignment was performed for the C-terminal region of Tb927.10.7910, the orthologues, and Z-beta (Zβ) of ZβADAR1, ZβDLM-1, and ZβPKZ domains.

### Cloning of a cDNA encoding full-length Tb927.10.7910 and creation of point mutations

The ORF of Tb927.10.7910 lacking the N-terminal mitochondrial import signal (the first eight amino acids: MFSSVLLR, as predicted by the Target IP4.1 server)^66^ was cloned into the pET30-a vector between NdeI and XhoI restriction sites to generate an N-terminal 6x His-tagged protein. The pET30-a/Tb927.10.7910 construct was used as the template to create nine individual alanine substitution point mutations. The point mutations located in the N-terminus of the protein included Thr52A, Arg53A, and Trp56A in the α3 region, Pro76A, Pro77A, and Trp79A in the wing region (β2-strand-loop-β3-strand) and Pro164A, Phe167A, and Trp188A in the C-terminal domain. All mutants were prepared by GenScript Corporation (Piscataway, NJ).

### Purification of the recombinant protein

The pET30-a expression vector was transformed into the T7 Express *lysy/I*^*q*^ competent *E*. *coli* strain (New England Biolabs SITE), which was grown to a density of 0.6 OD before induction with 0.5 mM isopropyl β-D-1-thiogalactopyranoside (IPTG). Bacterial cultures were grown after induction for either 5 h at 30 °C or 8 h at 16 °C, and then collected by centrifugation at 8,000 x g for 15 min at 4 °C. The cell pellet from 1 L of induced culture was resuspended in 50 ml cold PBS (pH 7.2), 10% glycerol, and 1X protease inhibitor mixture (Roche Applied Science), and cells were lysed by sonication on ice for 5 min, followed by centrifugation at 16,000 x g for 15 min at 4 °C. The cleared lysate was applied to a column with 2 mL IMAC Nickel charged resin (Bio-Rad). Proteins were eluted with an increasing gradient of imidazole from 10 mM to 320 mM, prepared in cold PBS containing 10% glycerol. Eluted fractions were dialyzed against two changes of buffer (PBS with 10% glycerol). The dialyzed recombinant proteins were applied to an Amicon centrifugal filter device (Millipore) and concentrated to 1/5 of the starting volume.

The relative sizes of the recombinant proteins were examined using SDS-PAGE (Fig. 5B) using an anti-6x His tag antibody (631212, Clontech) and visualized using a VersaDoc instrument (Bio-Rad) while the concentrations were measured using Quantity One software (Bio-Rad).

### *In vitro* transcription and radiolabeling of RNAs

Purified PCR fragments of gA6[14] Δ16G were amplified from the previously described plasmid encoding gA6[14] Δ16G^6^, which specifies the first ES of the ATPase subunit 6 (A6) pre-mRNA. A Riboprobe System-T7-promega kit was used for *in vitro* transcription of 2 μg template DNA^22^. The CYb pre-mRNA (102 nt)^67^ and edited CYb mRNA were transcribed from BamHI linearized plasmid and synthetic DNA antisense template with a T7 promoter sequence, respectively, using a RiboMAX Express-T7-promega kit. Transcripts were either labeled with [α- ^32^P] UTP (Perkin Elmer) during transcription or were radiolabeled after transcription with [α- ^32^P] pCp at the 3′ end using T4 RNA ligase (New England Biolabs).

Unlabeled RNAs used in competition assays were synthesized from the DNA oligonucleotides listed in Table 1, in combination with a T7 promoter oligonucleotide. The 90-nt pBlueScript SK + (Stratagene) RNA was generated by *in vitro* transcription of the NotI linearized plasmid. The pre-edited A6U5 transcript template was PCR amplified from the plasmid containing its sequence and used in the *in vitro* transcription reaction containing the A6U5 pre-mRNA. All RNAs were purified on 9% polyacrylamide/7 M urea gels.

**Table 1:** Oligonucleotides used for RNA-binding assays in this study.

Uridylated non-guide RNA; 49-nt	AAAAAAAAAAAAAAATAGTGATATCGAATTCCTTAGTATGTATCTGGTACCCTATAGTGACTCCTATTA
CYb edited RNA	TAAAAAGACAACATAAATTTCTAAATAATAAAAAAAATAACAAAAATCTAACACGAAAAAACATATTTCCCTATAGTGAGTCGTATTA
Poly AU RNA	AATAAGAGAGAAAAAATAAATAAATAAATAAATAAAAGAGACTCGAAAAGAATCCCTATAGTGAGTCGTATTA
Poly AU mutant RNA	AATAAGAGAGAAAAAATAGATAGATAGATAGATAAAAGAGACTCGAAAAGAATCCCTATAGTGAGTCGTATTA
42-mer CYb guide RNA	AAAAGCGGAGAAAAATTCACATTTCTTTTAATGTCAGTCCCCCCTATAGTGAGTCGTATTA
Natural guide CYb RNA	AAAAAAAAAAAAAAAATTATTCCCTTTATTACCTTAAGAAATTCACATTGTCTTTTAATCCCTATAGTGAGTCGTATTA
ga6(14) gRNA without U-tail	AATAATTATCATATCACTGTCAAAATCTGATTCGTTATGGAGTTATAGTATATTCCCCCTATAGTGAGTCGTATTA

Underlined sequences represent T7 promoter sequence.

### Gel shift assays

The apparent equilibrium dissociation constant (Kd app) was calculated for each RNA substrate by performing EMSAs^68^. For estimating Kd, increasing concentrations of purified RBP7910 (wild-type and point mutations) proteins were incubated with fixed concentrations of the labeled RNA (gA6[14] substrate and pre- and edited CYb mRNAs). For the gel shift assays, labeled RNAs were heated at 75 °C for 3 min followed by a slow cooling period with a rate of 1 °C/min to 23 °C, and held for 30 min at 23 °C before transferring the RNAs to the ice. Binding reactions were conducted in RBB50 buffer (20 mM Tris-HCl, pH 7.6, 50 mM KCl, 5 mM MgCl_2_, 100 mg/mL BSA, 10% glycerol, and 1 mM DTT), 100 mM KCl, and 20 units RNasin (Promega) in a 20 μl volume for 30 min at RT. Samples were mixed with gel loading dye (0.25% bromophenol blue, 0.25% xylene cyanol, and 30% glycerol) before loading onto native 10% TBE gels that were pre-run at 110 V for 15 min in 0.5 X TBE at 4 °C. After 2 h, gels were fixed with 10% isopropanol plus 7% acetic acid for 30 min and visualized using a PhosphorImager (Bio-Rad). Free and bound RNAs were quantified using Quantity One software (Bio-Rad). The sum of the bound complexes in each lane was considered the total bound fraction. Data were analyzed with nonlinear curve fitting methods using GraphPad Prism 7 software (GraphPad Software, Inc.). The values of Kd app and active protein concentrations, Bmax, were determined as best fits to the experimental data. The obtained Kd app values were used to calculate the active protein concentration and the corrected equilibrium dissociation constant using increasing concentrations of labeled RNAs relative to a fixed concentration of protein (wild-type and point mutants). The protein concentration was equivalent to approximately two times the estimated Kd app values.

Competition experiments were performed as described above using a fixed amount of protein that resulted in approximately 30–50% bound RNA. A saturating concentration of the radiolabeled gA6[14], CYb pre-mRNA, edited CYb mRNA, and AU target substrate was used in separate binding reactions and mixed with 1-, 10-, 100-, and 1000-fold molar excess concentrations of unlabeled competitor RNA in the RBB50 binding buffer prior to addition of the protein. Percent competition was estimated as the ratio of bound RNA in the presence of unlabeled competitor relative to RNA bound in the absence of competitor.

### Guanylyl transferase assay

RNA was isolated from (−Tet) and (+Tet) PF Tb927.10.7910 RNAi cells 3 and 4 days after Tet induction, and treated with DNase as described above. Eight micrograms of DNase-treated RNA were labeled with 10 μCi [α-^32^P] of GTP (3000 Ci/mmol) using a ScriptCap™ m7G Capping System kit (CELLSCRIPT™), according to the manufacturer’s instructions. Reactions were extracted with phenol: chloroform twice and chloroform once and precipitated. Samples were mixed with 80% formamide loading buffer and resolved on 8% acrylamide-7 M urea gel in 1 X TBE.

## Data Availability

The datasets are available from the corresponding author.
